# Omalizumab Reduces Unplanned Healthcare Interactions in Irish Patients With Chronic Spontaneous Urticaria

**DOI:** 10.3389/falgy.2021.810418

**Published:** 2021-12-23

**Authors:** Katie Ridge, Vyanka Redenbaugh, Niall Conlon

**Affiliations:** ^1^Clinical and Diagnostic Immunology, St. James's Hospital, Dublin, Ireland; ^2^Department of Clinical Immunology, School of Medicine, Trinity College Dublin, Dublin, Ireland

**Keywords:** chronic spontaneous urticaria, omalizumab, healthcare economics, dermatology specialty medicine, medicines access

## Abstract

Chronic spontaneous urticaria (CSU) is a common, debilitating skin disorder associated with impaired quality of life and psychological comorbidity. Symptoms can be difficult to control and many individuals will not respond to first line treatment. Due to the chronic and unpredictable nature of the disorder, patients frequently have repeated healthcare attendances. Despite this, little is known about healthcare resource utilization internationally. Furthermore, there is no Irish data to inform fundholding decision makers. Omalizumab is an anti IgE monoclonal antibody used in refractory urticaria. It is a comparatively high cost medicine and access to this treatment can be challenging. Recent assessments of omalizumab compared with usual care suggest that omalizumab is a cost-effective treatment for refractory urticaria. We carried out a retrospective review of 47 patients commenced on omalizumab. We evaluated unplanned primary and secondary care attendances and urticaria symptomatology before and after treatment. As expected, patients with refractory disease that were commenced on omalizumab had objective improvements in urticaria symptoms. Importantly, we show that this is reflected in a dramatic reduction in unplanned healthcare interactions at primary care and emergency departments. These data suggest that omalizumab may benefit these patients by reducing disease activity and thereby reducing the need for unplanned healthcare interactions.

## Introduction

Chronic spontaneous urticaria (CSU) is a condition characterized by recurring episodes of wheals lasting longer than 6 weeks. Angioedema can also be a feature. CSU has a major impact upon health-related quality of life, sleep, and daily activities (1). Psychological comorbidity is common and the persistent and unpredictable nature of the disorder results in significant health care access often with repeat attendances (2, 3). Time to diagnosis is prolonged. In Europe, the mean time to diagnosis is 2–4 years (4). The first-line symptomatic treatment for CSU is second generation antihistamines (5). However, up to 40% of patients will not respond to first-line treatment even when prescribed up to four times per day (6). Omalizumab is a safe and effective anti IgE monoclonal antibody that is recommended in CSU that is unresponsive to high dose second generation antihistamines (5). It is a high cost medicine that typically requires high level funding approval often with in-hospital administration and monitoring, thus demanding administrative and clinical support. In Ireland, access to omalizumab remains challenging with funding allocated on a case by case basis.

A recent study in the Netherlands which compared omalizumab for CSU with usual care found that omalizumab was a cost effective treatment in the Dutch setting (7). Among Dutch patients, indirect healthcare costs, particularly productivity costs, significantly contributed toward the drugs cost effectiveness. However, the transferability of cost effectiveness evaluations between countries is challenging and therefore local data is vital.

The aim of this study was to perform a retrospective single site review of patients commenced on omalizumab for refractory urticaria. We investigated unplanned healthcare attendances at primary and secondary care before and after treatment with omalizumab. We also assessed urticaria symptomatology before and after treatment with omalizumab.

## Methods

### Design

This retrospective single site study sought to assess the effectiveness of omalizumab treatment in CSU in relation to urticaria symptomatology and unplanned healthcare visits at a large teaching hospital in Ireland. Data was collected in the pre-pandemic era. Participants gave their informed consent to be involved in the study. The study dataset was collected by one clinical team member from patient clinical records; both written and electronic. Institutional ethics committee approval was in place from the SJH/TUH JREC approval number JREC 2017(03)CA17.

### Outcomes

We collected baseline data on participants including age, clinical diagnosis and current medication use.

#### Healthcare Attendances

Participants were asked whether they had made unplanned visits to the emergency department as a result of their urticaria or angioedema within the last 24 months and how many times they attended. Participants were also asked whether they had had any unplanned visits to their GP in the last 24 months. An unplanned GP visit was defined as an appointment sought for urgent or immediate management of the patient's urticaria or angioedema. Follow up data were collected between four and six months after initiation of omalizumab.

#### Urticaria Symptomatology

Participants receiving omalizumab for CSU (*n* = 42) completed the Urticaria Activity Score 7 which assesses wheals and itch over one week prior to commencing omalizumab (8). In addition, the urticaria control test (UCT) was used as a measure of urticaria symptomatology (9). This is a four item questionnaire whereby lower scores are indicative of higher symptom burden and was completed both pre and post initiation of omalizumab.

#### Adverse Effects and Subjective Improvement

Participants were asked to report any adverse effects 6 months after initiation of omalizumab. Participants were also asked whether or not they experienced a subjective improvement in their urticaria since commencing omalizumab.

## Results

Baseline characteristics are shown in Table 1. Females accounted for 78.7% (37/47) of participants. Chronic spontaneous urticaria that was refractory to high dose antihistamines was the most frequent indication for omalizumab (89.3%). Other indications for omalizumab included delayed pressure angioedema, recurrent spontaneous angioedema and symptomatic dermographism. The mean UAS7 score for patients with CSU was 35.4 (SD 5.05) whereby scores >28 suggest severe urticaria. The mean UCT score for patients with CSU prior to omalizumab was 2.5 (SD 1.8) where scores <12 suggest high disease activity and poor disease control.

**Table 1:** Baseline characteristics, *n* = 47.

Mean age	45.13 (SD 11.5)
Female gender	78.7%
CSUA	42/47 (89.3%)
Rescue steroid use in 12 months prior	37/47 (76%)
High dose antihistamine use	47/47 (100%)
Other immunosuppressant use	2/47 (4.3%)
Leukotriene antagonist use	33/47 (70.2%)
UAS7 at inclusion (*n =* 42)	35.4 (SD 5.05)
UCT at inclusion (*n =* 42)	2.5 (SD 1.8)
Unplanned ED attendances in 24 months, % participants	55.3%
Average number of ED attendances related to urticaria/angioedema per participant	1.3
Unplanned GP attendances in 24 months, % participants	91.5%
Average number of GP attendances related to urticaria/angioedema per participant	3.9

### Management of CSU

All participants were taking high dose antihistamines prior to commencing omalizumab. Leukotriene receptor antagonists were prescribed for 33 participants. The majority of patients had used rescue oral steroid therapy in the 12 months prior to commencing omalizumab. Two participants were taking other immunosuppressants (hydroxychloroquine, and methotrexate).

### Healthcare Attendances

The majority of participants (26/47) had attended the emergency department on at least one occasion in the preceding 24 months for urgent management of their symptoms. The majority of participants (43/47) had also attended their general practitioner (GP) for urgent management of their symptoms and the average number of GP attendances related to urticaria or angioedema per participant was 3.9.

### Pre and Post Omalizumab Assessments

As detailed in Table 2, unplanned hospital attendances related to urticaria or angioedema occurred in 26/47 participants in the preceding 24 months, with a cumulative total of 62 unplanned emergency department visits across all participants. After initiation of omalizumab, one participant out of 47 reported a single unplanned ED attendance. A comparison of unplanned hospital attendances before and after omalizumab showed that visits fell from 2.58 per month to 0.17 per month.

**Table 2:** Pre and post omalizumab assessments.

**Measure**	**Pre omalizumab**	**Post omalizumab (6 months)**
UCT score (*n =* 42)	2.5	13.1 (*p* < 0.00001)
Unplanned ED attendance, % of participants	55.3%	2%
Unplanned ED attendance, cumulative number of visits	62	1
Unplanned GP attendance, % of participants	91.5%	8.5%
Unplanned GP attendance, cumulative number of visits	181	4
Adverse effects	6.4%	
Subjective improvement in symptoms	97.9%	

Unplanned GP attendances related to urticaria or angioedema occurred in 43/47 participants in the preceding 24 months, with a cumulative total of 181 unplanned GP visits across all participants. After initiation of omalizumab, four participants out of 47 reported a cumulative eight GP attendances. Unplanned GP attendances before and after omalizumab fell from 7.54 visits per month to 1.33 visits per month.

The mean UCT score prior to the initiation of omalizumab was 2.5 (SD 1.8) suggestive of poorly controlled urticaria. Symptoms of urticaria reduced after initiation of omalizumab with a mean UCT score of 13.1 (SD 2.6) suggestive of low disease activity and good symptomatic control.

An adverse effect attributed to omalizumab was recorded for three participants. The adverse effects were subjective fluid retention, post injection headache and burning at injection site. A subjective improvement in symptoms after initiation of omalizumab was reported by 46/47 participants.

## Discussion

CSU is a common disease that affects all age groups (10). Patients with CSU frequently wait prolonged periods of time for an accurate diagnosis and refractory disease is frequent (4). Direct costs from CSU are high and are primarily related to pharmacological treatments as well as cost of hospitalization (1, 11). In addition, loss of productivity, absenteeism and impaired quality of life are well acknowledged in this cohort and represent indirect costs of disease management (1).

This retrospective review sought to describe unplanned healthcare interactions and urticaria symptomatology in patients with CSU before and after treatment with omalizumab. The demographics and characteristics of Irish patients included in our analysis were comparable to the data for the global cohort and from other studies (10). Results indicate that Irish patients with chronic spontaneous urticaria had more unplanned healthcare visits when their urticaria was symptomatic. In addition, the majority of patients reported rescue use of oral steroids. Six months after treatment with omalizumab, urticaria symptomatology improved and the number of unplanned healthcare visits fell. Reported adverse events were rare and almost all participants reported a subjective improvement in their symptoms upon initiation of omalizumab.

Calculation of primary care costs for this cohort is challenging in an Irish setting as universal primary care is not established. However, this study observed a 97.8% decrease in unplanned primary care attendance after omalizumab was initiated. With regards to secondary care, the unit cost of an emergency department visit in Ireland is €298 (12). The cost of secondary care as quantified by unplanned ED attendances fell from a cumulative figure of €18,476 to €298 after the initiation of omalizumab.

The cost of omalizumab includes drug acquisition at approximately €372.58 per month, as well as drug administration and monitoring in an ambulatory care setting (13). While it is apparent that patients' clinical improvement may lead to savings in indirect costs as observed in other cohorts, the cost effectiveness of omalizumab in an Irish setting remains unclear (7). A recent move toward self-administration of omalizumab offers an interesting approach toward streamlining services for CSU patients (14, 15).

It is noteworthy that the number of emergency department visits among CSU patients in the current study appear high, when compared with those reported in other countries (11). This suggests high direct costs of CSU management in Ireland. Recent data from the Netherlands propose that omalizumab is a cost effective treatment for the management of CSU when compared with usual care (7). In the Dutch setting, productivity costs, which comprised reduced numbers of hours at work and reduced efficiency at work were found to be key contributors to the cost effectiveness of omalizumab. Further characterization of healthcare use in CSU patients in Ireland will enable a rigorous assessment of cost-effective treatments.

The reduction of healthcare interactions in this study may be attributable to multiple factors. Omalizumab is an effective treatment for refractory urticaria that reduces symptom burden (5). However, patients commenced on omalizumab may also benefit from being linked into a specialist service, leading to targeted disease management and a more direct pathway to expert care. Patients who undergo specialist review are more likely to have been prescribed treatments that are in keeping with international urticaria guidelines (16). Furthermore, patients with access to specialist services may have the opportunity to contact a member of their clinical team when required, as opposed to making unscheduled visits to the emergency department or their GP. The natural course of CSU can have a relapsing and remitting pattern therefore spontaneous resolution of symptoms may also occur. Despite these points, we propose that the decrease in healthcare attendances and improvement in urticaria symptomatology in this patient group is noteworthy.

Our study is of value in that it is the first assessment of healthcare utilization in patients with CSU in Ireland. Findings are particularly useful to funding bodies who continue to evaluate the cost effectiveness of omalizumab for CSU. However, the sample size of the current study is small, and data were collected retrospectively. In addition, this study did not assess indirect costs of CSU management in Ireland which warrants further investigation.

## Conclusion

Although chronic spontaneous urticaria represents the most common cause for referral to clinical immunologists in Ireland, the prevalence and burden of this disease in the Irish setting is not well understood (17). This study demonstrates the high frequency of unplanned healthcare interaction when patients' CSU is active. Treatment with omalizumab resulted in a fall in urticaria symptomatology and reduced unplanned healthcare attendance. Patients who experience a clinical improvement in their CSU may indeed have improved productivity leading to reduced indirect costs of their disease. However, the direct cost of omalizumab is considerable and typically requires monitoring in ambulatory care. The transition toward self-administration of omalizumab provides an opportunity for understanding how the delivery of this treatment can be optimized in an Irish setting (15). While these data will be useful in informing funding decisions, further information on healthcare utilization in cohorts of patients with CSU across international jurisdictions is required.

## Data Availability Statement

The raw data supporting the conclusions of this article will be made available by the authors, without undue reservation.

## Ethics Statement

The studies involving human participants were reviewed and approved by St. James's Hospital and Tallaght University Hospital Joint Research Ethics Committee. The patients/participants provided their written informed consent to participate in this study.

## Author Contributions

KR wrote the manuscript. VR collected the data. NC conceived the analysis. All authors contributed to the article and approved the submitted version.

## Funding

This work was performed within the Irish Clinical Academic Training (ICAT) Programme, supported by the Wellcome Trust and the Health Research Board (Grant Number 203930/B/16/Z) and the Health Service Executive, National Doctors Training and Planning and the Health and Social Care, Research and Development Division, Northern Ireland.

## Conflict of Interest

The authors declare that the research was conducted in the absence of any commercial or financial relationships that could be construed as a potential conflict of interest.

## Publisher's Note

All claims expressed in this article are solely those of the authors and do not necessarily represent those of their affiliated organizations, or those of the publisher, the editors and the reviewers. Any product that may be evaluated in this article, or claim that may be made by its manufacturer, is not guaranteed or endorsed by the publisher.
